# SPATA33 is an autophagy mediator for cargo selectivity in germline mitophagy

**DOI:** 10.1038/s41418-020-00638-2

**Published:** 2020-10-21

**Authors:** Ying Zhang, Xu Xu, Mengxin Hu, Xin Wang, Hanhua Cheng, Rongjia Zhou

**Affiliations:** 1 Hubei Key Laboratory of Cell Homeostasis, College of Life Sciences, Wuhan, China; 2 Renmin Hospital of Wuhan University, Wuhan University, 430072 Wuhan, China; 3 Present Address: Luoyang Normal University, Henan, China

**Keywords:** Macroautophagy, Development

## Abstract

Selective autophagic degradation of mitochondria (mitophagy) is important in maintaining proper cellular homeostasis. Here, we found that SPATA33 is a novel autophagy mediator for mitophagy in testis. The SPATA33 protein localizes on mitochondria via its binding of the carboxyl terminal with the outer mitochondrial membrane protein VDAC2. Upon starvation induction, SPATA33 is recruited to autophagosome by binding the autophagy machinery ATG16L1 via its N-terminal along with mitochondria. Notably, *Spata33* knockout inhibited autophagy and overexpression can promote autophagosome formation for mitochondrial sequestration. Therefore, SPATA33 confers selectivity for mitochondrial degradation and promotes mitophagy in male germline cells.

## Introduction

Spermatogenesis is an orderly developmental process, which occurs within seminiferous tubules of the testis. It is a spatio-temporal event whereby undifferentiated spermatogonial germ cells with 2*n* chromosomes become spermatozoa with half number of chromosomes from spermatogonia, spermatocytes, and spermatids by proliferation, meiosis, and differentiation over a period of several weeks [1]. These processes are highly regulated to maintain cellular homeostasis by renewal and degradation of organelles and macromolecules, in which autophagy plays an important role [2, 3].

Autophagy is a cellular process of catabolism within cells, by which undesired cellular organelles and protein aggregates are degraded through autophagosome–lysosome pathway. Mitophagy, as a mitochondrion-specific autophagy, mediates the selective removal of damaged mitochondria [4]. Mitochondria are important organelles that provide energy, regulate programmed cell death and generate reactive oxygen species, and they are also crucial for the functioning of spermatogenesis. Mitophagy, as a cellular protective mechanism, can maintain the quantity and stability of mitochondria. Dysregulations of mitophagy were associated with many human diseases, for example, Parkinson’s disease [5], neuroprotection [6], chronic obstructive pulmonary disease [7], cardiac ischemia–reperfusion injury [8], and diabetic kidney disease [9].

Autophagy is active during spermatogenesis. There are some studies demonstrating the effects of autophagy on spermatogenic cells, including spermatogonia stem cells [10, 11], spermatogonia [12], spermatocytes [13, 14], and spermatozoa [15, 16]. Protein profiling of spermatogenic cells has identified several proteins in mice with high homology to the yeast autophagy related gene proteins (ATGs) [17, 18]. Some of these autophagy related proteins were essential for spermatogenesis. Knockout (KO) of *Atg5* and *Atg7* led to loss of testosterone production in Leydig cells in mice [19]. Abnormal acrosome biogenesis in *Atg7* and *Tbc1d20* KO mice [20, 21], meiotic initiation arrest in *Stra8* KO mice [22], the cytoskeletal disorganization in Sertoli cells in *Atg5* and *Atg7* KO mice [23], and impaired spermatid differentiation in *Atg7* KO mice [24] have also observed. These mutations eventually caused male infertility.

Several proteins and related pathways in regulation of mitophagy have been identified. The PINK1-PRKN pathway was involved in the regulation of mitophagy for eliminating damaged mitochondria in Parkinson’s disease [25]. Within this pathway, mitochondrial protein kinase PINK1 accumulates on damaged mitochondria, recruits and activates PRKN which ubiquitylates mitochondrial proteins. Meanwhile, PRKN activation is also accompanied by its autoubiquitination [26]. Two cytosolic autophagy receptors, NDP52 and OPTN, can recognize ubiquitinated mitochondria via their ubiquitin-binding domains, which also have LIR motif required to bind to LC3B on autophagic membranes [27, 28]. In addition, PINK1-mediated phospho-ubiquitin can amplify autophagic signals on damaged mitochondria [28]. These processes eventually lead to mitophagy to clean the damaged mitochondria. Insufficient mitophagy triggers accumulation of damaged mitochondria with stabilized PINK1, which was also associated with disease onset, such as chronic obstructive pulmonary disease pathogenesis [29].

PTENα was a key factor in cardiac protection via mitochondrial quality control. PTENα can recruit PRKN onto depolarized mitochondria through protein interaction for mitophagy [30]. Meanwhile, deubiquitinating enzymes can suppress these ubiquitination processes. For example, USP8 can deubiquitinate PRKN [26], while USP30 and USP35 can delay PRKN-mediated mitophagy [31]. Thus, deubiquitination functions as a balancing power in regulation of mitophagy. In addition, there are other receptor proteins that are not directly dependent on PARK2. For example, the mitochondrial E3 ligase MARCH5, but not PRKN, can ubiquitylate and degrade mitophagy receptor FUNDC1 in regulating hypoxia-induced mitophagy [32]. Deficiency of FUNDC1 was also associated with metabolic disorders [33]. However, mitophagy can occur in a ubiquitin-independent manner. In yeast, Atg32, a protein in the outer mitochondrial membrane, functions as an autophagic receptor through its interaction with Atg8 via its AIM-motif, and with Atg11 via its Atg11-binding domain for mitophagy [34, 35]. Atg11 acts as a scaffold protein to recruit Atg1 for autophagy initiation [36]. Although lack of Atg32 in mammals, the outer mitochondrial membrane proteins, BCL2L13 [37], BNIP3 [38], BNIP3L/NIX [39, 40], and FKBP8 [41], FUNDC1 [42], as well as inner mitochondrial membrane protein, PHB2 [43, 44], serve as functions of autophagy receptors similar to Atg32.

In addition to degradation of damaged mitochondria, elimination of needless or nondamaged mitochondria occurs as a critical quantity control mechanism for maintaining the proper amount of mitochondria [45]. Paternal mitochondria removal in zygote is a key step to ensure maternal inheritance of mitochondria. Both ubiquitin proteasome system and sperm mitophagy occurred during the elimination process [46–49]. Studies in the porcine zygote suggested that a combined action of SQSTM1-dependent autophagy and VCP-mediated ubiquitination of sperm mitochondrial proteins was responsible for sperm mitophagy [47, 48]. In *Caenorhabditis elegans*, LC3 was a key regulator to control the fate of sperm mitochondria via autophagosome targeted to the pericentrosomal area [50]. Recent study showed that FNDC-1 was a mitophagy receptor and essential for paternal mitochondria elimination in *C. elegans* [51].

Despite these mitophagy receptors being characterized, tissue or cell-type specific receptors for mitophagy and their precise mechanisms of recognition and degradation are still unclear. We have previously identified a novel gene *Spata33*, also called as *4732415M23Rik* or *C16orf55*, which is conserved in mammals and specifically expressed in mouse testis [52]. Pathological analysis showed that SPATA33 was decreased in testis of spermatozoa deficient patients [53]. In this study, we report that SPATA33 functions as autophagy mediator as well as its roles in promoting mitophagy in germline. SPATA33 is a novel autophagy mediator for mitophagy through directly binding to the autophagy machinery ATG16L1 and the outer mitochondrial membrane protein VDAC2. This characteristic confers the cargo selectivity during mitophagy.

## Results

### SPATA33 is colocalized with autophagy proteins associated with mitochondria of mouse germline cells

**Fig. 1:**
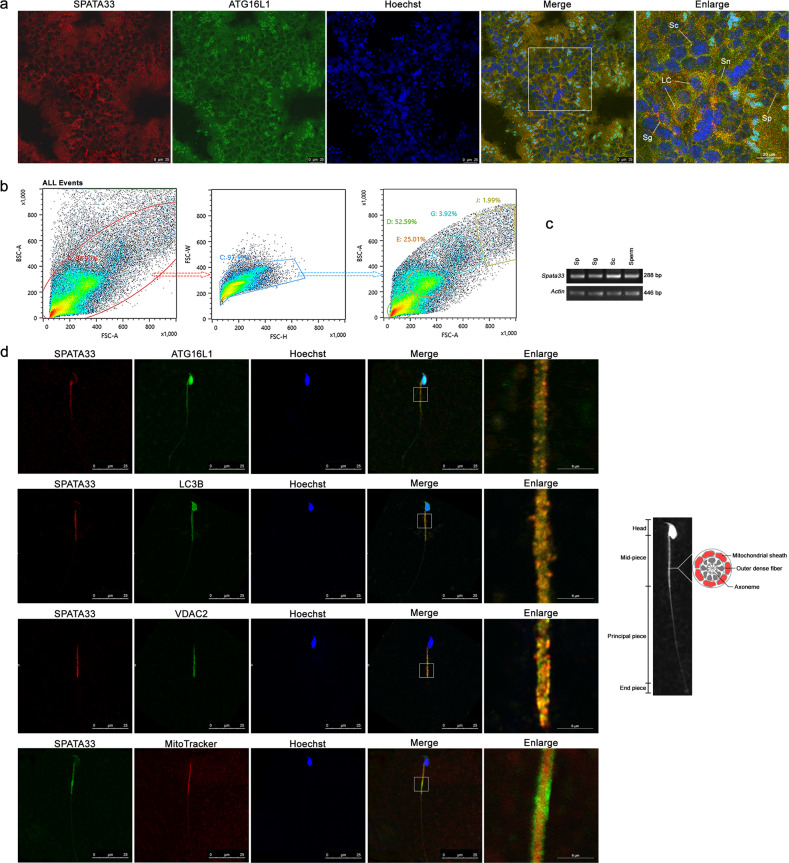
SPATA33 and ATG16L1 are colocalized in germline cells in mice. **a** Immunofluorescence of SPATA33 and ATG16L1 proteins in mouse testis. Anti-SPATA33, anti-ATG16L1 (Alexa Fluor^®^488), and TRITC-conjugated goat anti-rabbit IgG (H + L) antibodies were used to detect SPATA33 (red) and ATG16L1 (green). The nuclei were stained by Hoechst (blue). Images were taken by confocal fluorescence microscopy (SP8, Leica, Wetzlar, Germany). Positive signals were detected in Leydig cells (LC), Sertoli cells (Sn), spermatogonia (Sg), spermatocytes (Sc), and spermatids (Sp). Scale bar, 25 μm; scale bar in enlarged panels: 20 μm. **b** Cell sorting of spermatogonia (Sg), spermatocytes (Sc), and the round spermatids (Sp) from testis by cell flow sorter. Serial charts of cells were indicated. **c** Expression of *Spata33* was detected in spermatogonia (Sg), spermatocyte (Sc), the round spermatids (Sp), and spermatozoa (sperm). The sperm cells were isolated from epididymis of adult mouse. RT-PCR was performed from mRNAs isolated from these cells. *Actin* was used as an internal control. **d** SPATA33 was colocalized with ATG16L1, LC3B, and VDAC2 in the mid-piece region (mitochondria region) of the sperm cells. The spermatozoa were extracted from epididymis of adult mice. The cells were stained with MitoTracker (mitochondria matrix marker, red). Immunofluorescence analysis were performed with anti-SPATA33, anti-ATG16L1 (Alexa Fluor^®^488), anti-VDAC2, anti-LC3B (Alexa Fluor^®^488), TRITC-conjugated goat anti-rabbit IgG (H + L), and FITC-conjugated rabbit anti-goat IgG (H + L) antibodies. The enlarged images were originated from the squares in the merged panels. The nuclei were stained by Hoechst (blue). Images were taken by confocal fluorescence microscopy (SP8, Leica). The graph on the right indicates sperm ultrastructure, highlighting cross-section of the mid-piece region, including axoneme, outer dense fiber, and mitochondrial sheath (red). Scale bar: 25 μm; scale bar in enlarged panels: 6 μm.

Our previous study indicated that the novel gene *Spata33* is evolutionarily conserved in mammals and associated with spermatogenesis [52]. In expression timing, SP1 precedes SPATA33 in postnatal testis, and it can specifically bind to the *Spata33* promoter region in vivo and activates its expression in testis (Fig. S1). To further explore functions of SPATA33 in the germline, we first analyzed its expression relationship with potential partners. Immunofluorescence analysis of SPATA33 protein in adult mouse testis showed a co-expression pattern with key autophagy protein ATG16L1 in the cytoplasm of three main types of cells, including Sertoli, Leydig, and spermatogenic cells (Fig. 1a). Cell sorting of testis samples and RT-PCR confirmed *Spata33* expression in spermatogonia, spermatocytes, round spermatids, and spermatozoa (Fig. 1b, c). Further sperm immunofluorescence indicated that SPATA33 protein was colocalized with key autophagy proteins ATG16L1 and LC3B, and mitochondrial outer membrane protein VDAC2 in the mid-piece region (mitochondria region) of spermatozoa from epididymis of adult mice (Fig. 1d). In addition, MitoTracker (a mitochondria matrix marker) co-staining indicated that SPATA33 protein was located in the mitochondrial sheath of the mid-piece region (Fig. 1d). These results suggested that SPATA33 is potentially associated with mitophagy in spermatogenesis.

### SPATA33 is associated with autophagy through interaction with ATG16L1

**Fig. 2:**
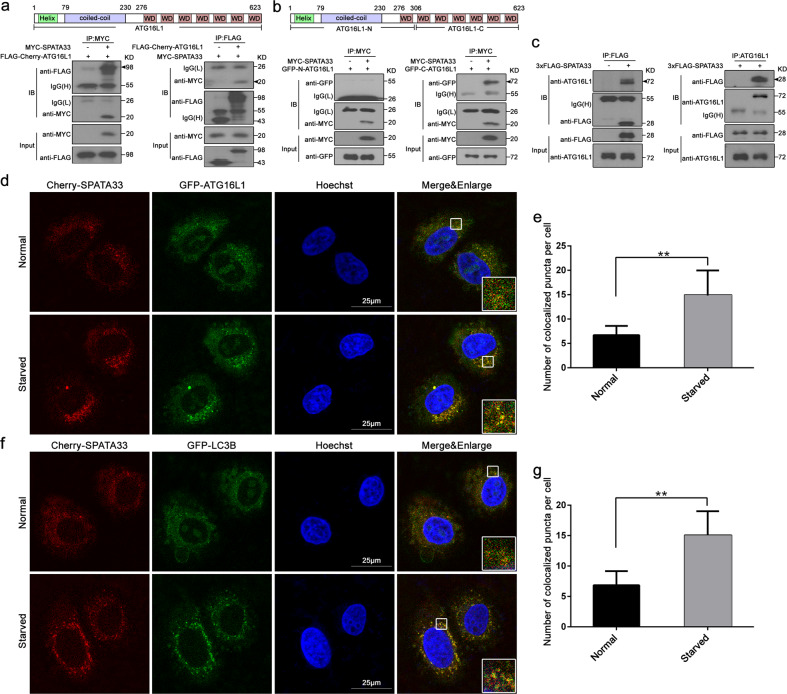
SPATA33 interacts with ATG16L1 to form autophagic puncta upon starvation induction. **a** Coimmunoprecipitation of MYC-SPATA33 with Cherry-FLAG-ATG16L1. HEK293T cells were transiently transfected with pMYC-SPATA33 and pCherry-FLAG-ATG16L1. After 48 h, the whole cell lysate was extracted for coimmunoprecipitation with anti-MYC, or anti-FLAG. Anti-FLAG or anti-MYC was also used for western blotting. Arrowheads indicate the immunoprecipitated bands. **b** Coimmunoprecipitation between MYC-SPATA33 and deletion mutants of ATG16L1. pMYC-SPATA33 was transiently co-transfected with pGFP-N-ATG16L1 or pGFP-C-ATG16L1 in HEK293T cells. Cell lysates were examined by western blotting using the anti-MYC or anti-GFP antibody. For coimmunoprecipitation, the lysates were immunoprecipitated with anti-MYC, followed by immunoblotting with the anti-GFP antibody. Arrowheads indicate the immunoprecipitated bands. **c** Endogenous ATG16L1 interacted with FLAG-SPATA33. HEK293T cells were transiently transfected with p3xFLAG-SPATA33, and after 48 h, the whole cell lysate was extracted for coimmunoprecipitation with anti-ATG16L1 or anti-FLAG. Anti-ATG16L1 or anti-FLAG antibody was also used for western blotting. **d** Colocalization of SPATA33 with ATG16L1 in HeLa cells under starvation condition. HeLa cells were transiently co-transfected with pCherry-SPATA33 and pGFP-ATG16L1. After cultured in normal medium for 24 h, the cells were starved in EBSS medium for 2 h, and analyzed by confocal microscopy. The nuclei were stained by Hoechst (blue). The insets showed an enlarged view of the indicated squares. Yellow puncta in merged panels are the colocalized puncta. Scale bar: 25 μm. **e** Statistical analysis of colocalized puncta between SPATA33 and ATG16L1. **f** Colocalization of SPATA33 with LC3B in HeLa cells under starvation condition. HeLa cells were transiently co-transfected with pCherry-SPATA33 and pGFP-LC3B and cultured in normal medium for 24 h. The cells were starved in EBSS medium for 2 h, and analyzed by confocal microscopy. The nuclei were stained by Hoechst (blue). The insets showed an enlarged view of the indicated squares. Yellow puncta in merged panel are the colocalized puncta. Scale bar: 25 μm. **g** Statistical analysis of colocalized puncta between SPATA33 and LC3B. Data are presented as means ± S.D. **p < 0.01 (*n* = 3 independent experiments).

To investigate potential molecular mechanisms of SPATA33 in autophagy, coimmunoprecipitation analysis was used to determine interaction of SPATA33 with ATG16L1. Coimmunoprecipitation with either anti-MYC or anti-FLAG after co-transfection of MYC-SPATA33 and FLAG-Cherry-ATG16L1 showed that SPATA33 can interact with ATG16L1 (Fig. 2a). Deletion analysis indicated that SPATA33 was bound to the C-terminus of ATG16L1, but not to the N-terminus of ATG16L1 (Fig. 2b). Coimmunoprecipitation experiment confirmed that SPATA33 can interact with endogenous ATG16L1 (Fig. 2c). Further fluorescence microscopy analysis showed that both SPATA33 and ATG16L1 were colocalized in HeLa cells, upon starvation induction in particular (Fig. 2d). Statistical analysis showed that number of colocalized puncta between SPATA33 and ATG16L1 was significantly higher under starvation condition in comparison with normal culture (Fig. 2e). Furthermore, SPATA33 had also obvious colocalization with key autophagy protein LC3B (Fig. 2f, g). These results indicated that SPATA33 was associated with autophagy through its interaction with ATG16L1.

### *Spata33* knockout suppresses mitophagy

**Fig. 3:**
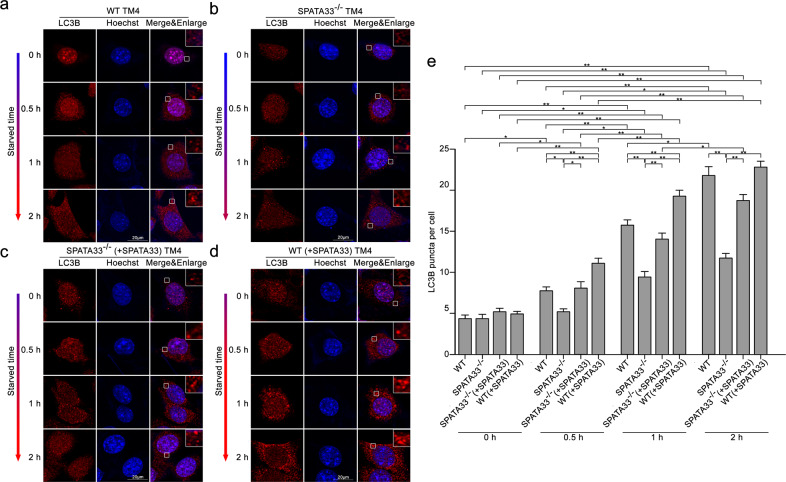
*Spata33* knockout inhibits autophagy. **a** LC3B puncta were detected in WT TM4 cells after starvation culture (EBSS) for 0.5 h. The cells were cultured in EBSS for the indicated time and analyzed by immunofluorescence with the anti-LC3B antibody and confocal microscopy. The insets showed an enlarged view of the indicated squares and highlighted red LC3B puncta in the cytoplasm. The nuclei were stained with Hoechst reagent. Scale bar: 20 μm. **b** LC3B puncta were detected at 1 and 2 h under starvation culture in the *Spata33*^−/−^ cells. Scale bar: 20 μm. **c** To rescue *Spata33* expression in the *Spata33*^−/−^ cells, the cells were infected with lentivirus expressing 3xFLAG-SPATA33. Immunofluorescence with the anti-LC3B antibody showed obvious LC3B puncta appeared again at 0.5 h under starvation culture. Scale bar: 20 μm. **d** To overexpress *Spata33* in the WT TM4 cells, the cells were infected with lentivirus expressing 3xFLAG-SPATA33 and analyzed by immunofluorescence with the anti-LC3B antibody. Obvious LC3B puncta were detected again at 0.5 h under starvation culture. Scale bar: 20 μm. **e**. Statistical analysis of LC3B puncta from (**a**) to (**d**). The number of LC3B puncta in 30 cells was counted for each group. Data are presented as mean ± S.D. *p < 0.05; **p < 0.01 (*n* = 3 independent experiments).

**Fig. 4:**
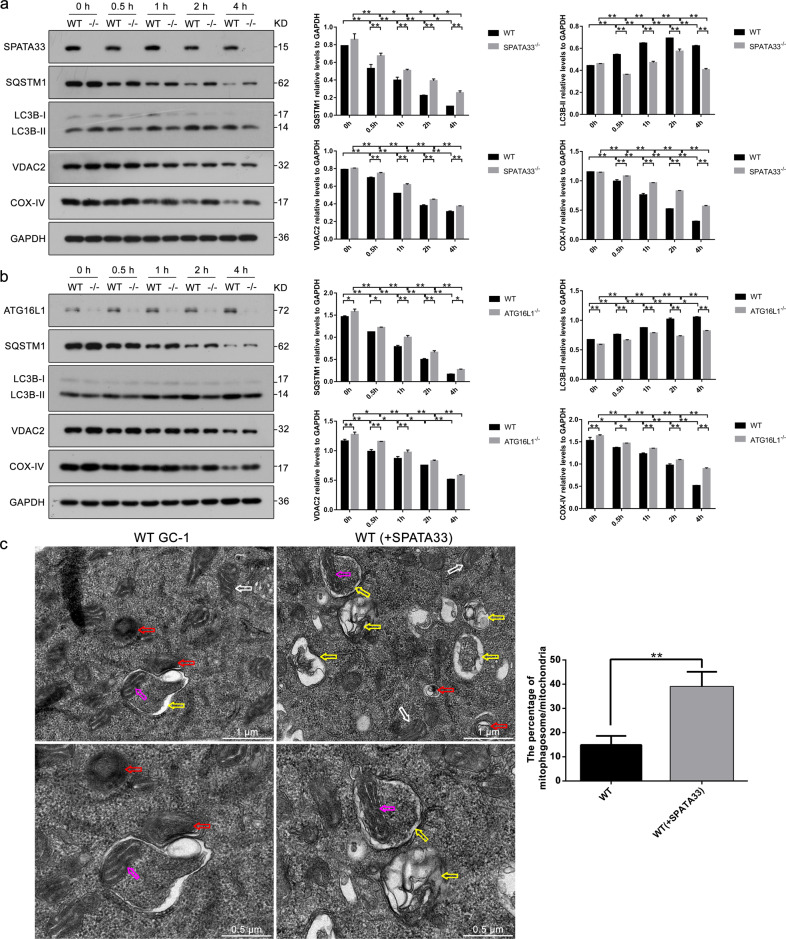
*Spata33* knockout inhibits mitophagy. **a** *Spata33* knockout decreased autophagy and mitochondrial related protein levels. WT TM4 and *Spata33*^−/−^ cell lines (#32-10) were cultured in the EBSS medium for 0, 0.5, 1, 2, and 4 h, respectively. Cell lysates were analyzed by immunoblotting with the anti-SQSTM1, anti-LC3B, anti-VDAC2, or anti-COX-IV. GAPDH was used as an endogenous control. The graphs on the right panels indicate statistical analysis of the gray scanned SQSTM1, LC3B, VDAC2, and COX-IV from (**a**). **b** *Atg16l1* knockout decreased autophagy and mitochondrial related protein levels. WT TM4 and *Atg16l1*^−/−^ cell lines (#1-13, the group were not from single KO clone) were cultured in the EBSS medium for 0, 0.5, 1, 2, and 4 h, respectively. Cell lysates were analyzed by immunoblotting with the anti-SQSTM1, anti-LC3B, anti-VDAC2, or anti-COX-IV antibody. GAPDH was used as an endogenous control. The graphs on the right panels indicate statistical analysis of the gray scanned SQSTM1, LC3B, VDAC2, and COX-IV from (**b**). **c** Electron microscopy showed the mitophagosome in wild type and SPATA33-overexpressed GC-1 cells. Enlarged images were showed in the lower panels. The yellow arrow indicates autolysosome, the purple arrows show the degraded mitochondria in autolysosome, the white arrow indicates the normal mitochondria, and the red arrow indicates lysosome. Scale bar: 1 μm, scale bar in enlarged panels: 0.5 μm. The graphs on the right indicate statistical analysis of the percentage of mitophagosome/all mitochondria. Data are presented as means ± S.D. **p < 0.01 (*n* = 3 independent experiments).

To further explore roles of SPATA33 in mitophagy, we constructed both *Spata33*^−/−^ TM4 (Sertoli cells) and GC-1 (spermatogenic cells) cell lines using CRISPR/Cas9 technology. Sequencing and off-target analysis showed that *Spata33* was efficiently KO in both cell lines (Figs. S2–S5). *Spata33*^−/−^ cells (#32-10) were further used to detect autophagosome formation under starvation condition. Immunofluorescence analysis showed that obvious LC3B puncta were detected at 1 h under starvation culture condition in *Spata33*^−/−^ cells, whereas LC3B puncta appeared at 0.5 h under starvation condition in wild-type cells (Fig. 3a, b, e). When *Spata33* expression was rescued in *Spata33*^−/−^ cells by infecting with lentivirus expressing *Spata33*, obvious LC3B puncta appeared again at 0.5 h under starvation condition (Fig. 3c, e). In *Spata33* overexpression in WT cells by infecting with lentivirus expressing *Spata33*, LC3B puncta were also observed at 0.5 h under starvation condition (Fig. 3d, e). These results showed that *Spata33* overexpression promoted formation of autophagosome, while *Spata33* KO inhibited the formation of autophagosome. Western blot analysis showed that LC3B-II, which is a lipidated form of the key autophagy protein LC3B-I, was significantly decreased in *Spata33*^−/−^ cells upon starvation induction in comparison with the normal culture condition (Fig. 4a). SQSTM1, as a substrate for autophagy degradation, had an opposite trend (Fig. 4a). At the same time, we also examined whether protein levels of mitochondrial outer membrane VDAC2 and inner membrane COX-IV were affected when autophagy decreased. Western blot analysis showed that starvation treatment resulted in an obvious decrease of degradation of VDAC2 and COX-IV in *Spata33* KO compared to wild type, indicating that mitochondria was an autophagic target (Fig. 4a). The *Atg16l1* gene KO cell lines were also constructed, and LC3B-II, SQSTM1, VDAC2, and COX-IV were detected by the same method. It was found that the *Atg16l1* KO showed a similar effect on autophagy inhibition as *Spata33* KO (Fig. 4b).

**Fig. 5:**
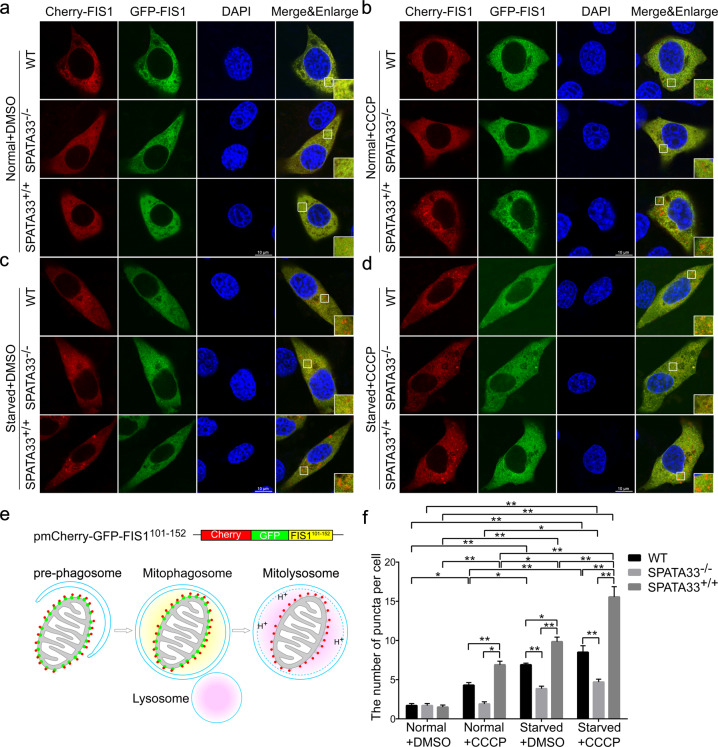
MitoQC-based analysis. **a**–**d** Fluorescent microscopy of the pmCherry-GFP-FIS1^101-152^ tandem reporter in *Spata33* knockout (*spata33*^−/−^, #33), *Spata33* overexpression (*spata33*^+/+^), and wild-type GC-1 cell lines. The cells were transfected with the pmCherry-GFP-FIS1^101-152^ and cultured in normal (control), CCCP (10 μM, 1 h), EBSS medium (1 h) or EBSS with CCCP (10 μM, 1 h) addition, respectively. Single channel (red, green, or blue) and merged images were taken by confocal microscopy. The insets showed an enlarged view of the indicated squares. Scale bar: 10 μm. **e** Schematic diagram of MitoQC-based analysis principle. pmCherry-GFP-FIS1^101-152^ tandem reporter expresses a tandem mCherry-GFP tag fused to the mitochondrial outer membrane protein FIS1, MitoQC displays red and green fluorescence during steady-state conditions, but the mCherry signals become stable when mitophagy is induced, because mitochondria are delivered to the lysosome where the GFP signals are quenched. Therefore, mCherry-only puncta are seen during mitophagy activation. **f** Statistical analysis of vesicles positive for mCherry puncta (mitolysosome) (>15 cells per experiment) by *t*-test in the (**a**–**d**). The mean ± SD are from three independent experiments. *p < 0.05; **p < 0.01.

In addition, we observed damaged mitochondria being engulfed by autophagosomes in *Spata33*-overexpressed GC-1 cell line by transmission electron microscope under combined treatment of starvation and CCCP (an inducer of mitophagy), in addition to autolysosomes, in which damaged mitochondria were being degraded (Fig. 4c). Statistical analysis showed that the level of mitochondrial autophagy in *Spata33*-overexpressed cells was significantly higher than that in wild-type cells (Fig. 4c). Furthermore, MitoQC-based analysis using the pmCherry-GFP-FIS1^101-152^ tandem reporter (FIS1, a mitochondrial outer membrane protein) in *Spata33* KO (*Spata33*^−/−^, #33), *Spata33* overexpression, and wild-type GC-1 cell lines showed that *Spata33* KO significantly decreased the level of mitophagy, while *Spata33* overexpression promoted mitophagy (Fig. 5a–f). Together, these results suggested that SPATA33 promoted mitophagy in germ cells.

### SPATA33-associated autophagy flux

**Fig. 6:**
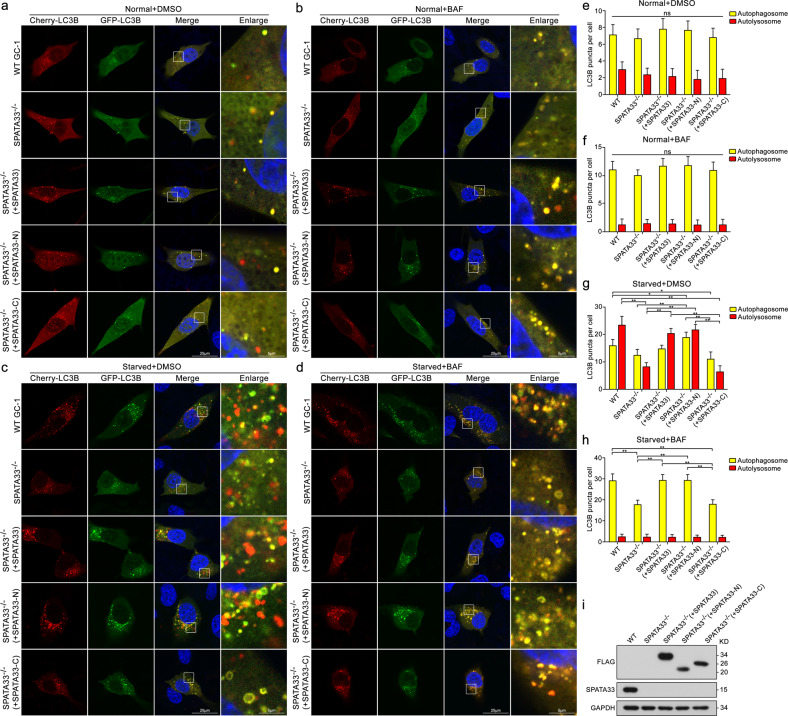
SPATA33-associated autophagy flux. **a**–**d** The pmCherry-GFP-LC3B tandem reporter analysis in *Spata33* knockout (*Spata33*^−/−^, #33), SPATA33 rescued (stably expressing SPATA33 in *Spata33*^−/−^ cell lines), SPATA33-N rescued (stably expressing SPATA33-N in *Spata33*^−/−^ cell lines), SPATA33-C rescued (stably expressing SPATA33-C in *Spata33*^−/−^ cell lines), and wild-type GC-1 cell lines. Representative images of the cells transfected with the pmCherry-GFP-LC3B reporter and cultured in normal (control), bafilomycin A1 (100 nM) addition (1 h), EBSS medium (1 h), or EBSS with bafilomycin A1 (100 nM) addition (1 h), respectively. Single channel (red, green or blue) and merged images were taken by confocal microscopy. Scale bar: 25 μm, scale bar in enlarged panels: 5 μm. **e–h** Statistical analysis of vesicles positive for both GFP and mCherry (autophagosomes) and for mCherry (autolysosomes) (>15 cells per experiment) by *t*-test in the (**a**–**d**), respectively. The mean ± SD are from three independent experiments. *p < 0.05; **p < 0.01. **i** Determination of effectively rescuing of SPATA33-N and SPATA33-C. Endogenous SPATA33, FLAG-SPATA33, FLAG-SPATA33-N, and FLAG-SPATA33-C protein levels were analyzed by western blotting.

To investigate SPATA33-associated autophagy process, we tested the SPATA33-involved autophagy flux through *Spata33* KO and forced expression. Autophagy flux tests were performed using a tandem fluorescent indicator, mCherry-GFP-LC3B, in *Spata33* KO (*Spata33*^−/−^, #33), SPATA33 rescued (stably expressing SPATA33 in *Spata33*^−/−^ cell line), SPATA33-N rescued (stably expressing SPATA33-N in *Spata33*^−/−^ cell lines), SPATA33-C rescued (stably expressing SPATA33-C in *Spata33*^−/−^ cell lines), and wild-type GC-1 cell lines. Since green fluorescence of the fusion protein is very sensitive to the acidic environment of lysosomes and quickly quenched in autolysosomes, just red fluorescence could be detected in autolysosomes. Fluorescence analysis using the tandem fluorescent indicator system in these cell lines showed that *Spata33* KO significantly inhibited formation of autophagosomes, while both rescues of SPATA33 and SPATA33-N promoted the formation of autophagosomes (Fig. 6a–d, i). Under normal conditions or bafilomycin A1 treatment, there was no significant difference between these cell lines (Fig. 6a, b, e, f). However, SPATA33-N rescue could promote the formation of autophagosome, while SPATA33-C could not rescue autophagy inhibition under starvation condition (Fig. 6c, g). This also confirmed that the interaction between SPATA33 and ATG16L1 was necessary to promote autophagy. Further starvation and bafilomycin A1 combined treatment showed a significant accumulation of autophagosomes (Fig. 6d, h). These results suggested that SPATA33 plays a role in autophagosome formation.

### SPATA33 mediates mitophagy through linking between VDAC2 and ATG16L1

**Fig. 7:**
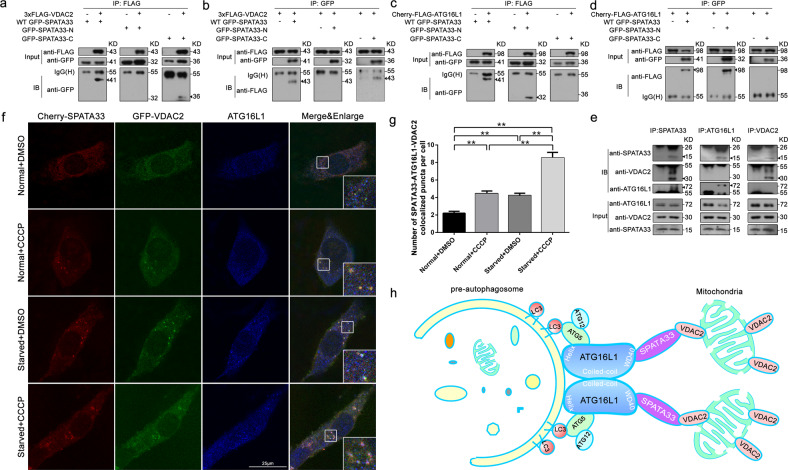
SPATA33 interaction with ATG16L1 and VDAC2. **a**, **b** Coimmunoprecipitation between VDAC2 and deletion mutants of SPATA33. pCherry-FLAG-ATG16L1 was transiently co-transfected with pGFP-SPATA33, pGFP-N-SPATA33, or pGFP-C-SPATA33 in HEK293T cells, respectively. Cell lysates were examined by western blotting using the anti-FLAG or anti-GFP antibody. For coimmunoprecipitation, the lysates were immunoprecipitated with anti-FLAG or anti-GFP, followed by immunoblotting with the anti-GFP or anti-FLAG antibody. Arrowheads indicate the target bands. **c**, **d** Coimmunoprecipitation between ATG16L1 and deletion mutants of SPATA33. p3xFLAG-VDAC2 was transiently co-transfected with pGFP-SPATA33, pGFP-N-SPATA33, or pGFP-C-SPATA33 in HEK293T cells, respectively. Cell lysates were examined by western blotting using the anti-FLAG or anti-GFP antibody. For coimmunoprecipitation, the lysates were immunoprecipitated with anti-FLAG or anti-GFP, followed by immunoblotting with the anti-GFP or anti-FLAG antibody. Arrowheads indicate the target bands. **e** Coimmunoprecipitation analysis of interaction among endogenous SPATA33, ATG16L1, and VDAC2 in GC-1 cells. The GC-1 cell lysates were immunoprecipitated with anti-SPATA33, anti-ATG16L1, or anti-VDAC2 antibody, followed by immunoblotting with the anti-SPATA33, anti-VDAC2, or anti-ATG16L1 antibody, respectively. The whole cell lysates were examined by western blotting using the anti-ATG16L1, anti-VDAC2, or anti-SPATA33 antibody. Arrowheads indicate the target bands. **f** Colocalization analysis of SPATA33 with VDAC2 and ATG16L1. GC-1 cells were transiently co-transfected with pCherry-SPATA33 and pGFP-VDAC2. After 24 h in normal medium, the cells were cultured in normal (control), CCCP (10 μM, 1 h), EBSS medium (1 h), or EBSS with CCCP (10 μM, 1 h) addition, respectively. Immunofluorescence analysis was performed with anti-ATG16L1 and Dylight 405 Donkey anti-Rabbit IgG (H + L) antibodies. Single channel (red, green, or blue) and merged images were taken by confocal microscopy. Colocalizing structures are indicated in white (merge). Scale bar: 25 μm. **g** Statistical analysis of colocalized puncta between SPATA33, ATG16L1, and VDAC2. Data are presented as means ± S.D. **p < 0.01 (*n* = 3 independent experiments, >15 cells per experiment). **h** SPATA33 mediates mitophagy via interaction with VDAC2 and ATG16L1. SPATA33 interacts with mitochondrial outer membrane protein VDAC2 through its carboxyl terminal, while its amino terminal interacts with WD40 region of ATG16L1 protein. Upon starvation stress, damaged mitochondria are encapsulated by autophagosome through mediator SPATA33 linking between VDAC2 and ATG16L1 to initiate mitophagy.

To further explore how SPATA33 mediates mitophagy, we analyzed SPATA33 interaction with the mitochondrial outer membrane protein VDAC2 by coimmunoprecipitation. We found that SPATA33 can interact with VDAC2 in the coimmunoprecipitation analysis (Fig. 7a). SPATA33 interacted via its C-terminus with VDAC2, but not to its N-terminus (Fig. 7a, b). Deletion mapping and coimmunoprecipitation showed that ATG16L1 bound to the N-terminus of SPATA33 (Fig. 7c, d), whereas VDAC2 interacted with the C-terminus of SPATA33. Endogenous interactions among SPATA33 and ATG16L1 or VDAC2 were confirmed in GC-1 cell lines (Fig. 7e). Fluorescence microscopy showed that there was an obvious colocalization between Cherry-SPATA33 and GFP-VDAC2 and ATG16L1, which were enhanced significantly by starvation and CCCP combined treatment (Fig. 7f, g). A similar result was also observed in HeLa cells (Fig. S6). Taken together, SPATA33 mediates mitophagy via its carboxyl terminal by interaction with the mitochondrial outer membrane protein VDAC2, while SPATA33 can also interact via its N-terminal with the WD40 region of ATG16L1 protein (Fig. 7h). Upon starvation stress, damaged mitochondria are encapsulated by pre-autophagosome through mediator SPATA33 linking between VDAC2 and ATG16L1 to initiate mitophagy.

## Discussion

Selective autophagy is highly regulated through cargo selectivity and autophagy receptors, relative to formerly nonselective bulk degradation pathway. Mitophagy, as a typic process of selective autophagic degradation of mitochondria, plays important roles in cellular quality control that eliminates damaged and superfluous mitochondria. Dysregulation of mitophagy has been associated with several human diseases, including neurodegenerative disorders such as Parkinson’s disease [54]. Despite the considerable efforts in identification of autophagy receptors and pathways involved in mitophagy, tissue or cell-type specific receptors for mitophagy and their precise mechanisms of recognition and degradation are still unclear. Here we identify SPATA33 as a novel autophagy mediator for mitophagy in germline. The autophagy mediator is particularly important, as it not only broadens our understanding of cellular quality control and mitochondrial homeostasis, but also has the implications of the SPATA33-mediated mitophagy in the germline development and diseases.

The autophagy mediator SPATA33 has several distinct features in mammalian mitophagy. In autophagy functions, it can promote mitophagy through interaction with ATG16L1 upon stress, such as starvation and mitochondrial damage, as well as being an autophagy mediator. Autophagy flux assays confirmed that it promoted the formation of autophagosomes, but not influenced autophagosome fusion with lysosome. In yeast, mitophagy is mediated by the outer mitochondrial membrane receptor Atg32, which links mitochondria to the phagophore by directly binding Atg8 via its AIM-motif (Atg8-interacting motif) [34]. Atg32 can also interact with scaffold protein Atg11, which eventually recruits Atg1 complexes to induce autophagy [36]. However, a counterpart to yeast Atg11 has yet to be discovered in mammals. In this study, we suggest that SPATA33 may exert roles as a bi-functional counterpart of both Atg32 and Atg11 in mammals. The difference is that SPATA33 can directly links damaged mitochondria to autophagosomes via its binding to the outer mitochondrial membrane protein VDAC2, as well as to key autophagy machinery ATG16L1. This characteristic probably confers an efficient and specific mechanism of cellular quality control through SPATA33-mediated mitophagy in mammalian germline.

In SPATA33-mediated mitophagy, cargo selectivity is provided by the autophagy protein SPATA33, which directly links targeted mitochondria via its binding to the outer mitochondrial membrane protein VDAC2 and to the autophagic machinery ATG16L1. The mitophagy process is probably ubiquitin independent. So far, all ubiquitin-independent autophagy receptors on the outer mitochondrial membrane (BCL2L13, BNIP3, NIX, FKBP8, FUNDC1, and Atg32) have a common LIR/AIM-motif, which can bind to LC3B/Atg8 on autophagic membranes [55]. Compared with these receptors, SPATA33 directly interacts with the autophagic machinery ATG16L1, instead of LC3B. Thus, SPATA33-mediated mitophagy probably occurs when ATG5-ATG12 recruits ATG16L1 to form ATG5-ATG12-ATG16L1 complex on autophagic membranes upon autophagy induction. In autophagy vesicle elongation, ATG12-ATG5-ATG16L1 complex assembly is a key event. The small GTPase RAB37 regulates autophagosome formation through a switch between the GTP-bound “on” and the GDP-bound “off” forms. RAB37-GTP interacts directly with ATG5 and promotes the interaction of ATG12-ATG5 with ATG16L1 [56, 57]. The RAB37-ATG12-ATG5-ATG16L1 complex eventually recruits and lipidates LC3B-I to form active LC3B-II, which accelerates autophagosome formation.

Tissue or cell-type specific mitophagy is important to degrade unnecessary or damaged mitochondria to maintain mitochondrial homeostasis in development and diseases. The SPATA33-mediated mitophagy is male germline specific, which could play important roles in spermatogenesis. The autophagy mediator SPATA33 is specifically expressed in spermatogenic cells, including spermatogonia, spermatocytes, round spermatids, and spermatozoa during spermatogenesis, in which key autophagy machinery ATG16L1 and LC3B, and mitochondrial outer membrane protein VDAC2 are also expressed. In addition, SP1 specifically binds to the *Spata33* promoter region in vivo and activates its expression during spermatogenesis. Notably, SPATA33 promotes mitophagy as well as being an autophagy mediator. These results support its role in mitophagy in the germline development and differentiation. As autophagy is indispensable for spermatogenesis [58], identification of the autophagy mediator SPATA33 in germline provides a new understanding of maintaining cellular homeostasis during spermatogenesis through mitophagy. In addition, paternal mitochondria removal in zygote is a key step to ensure maternal inheritance of mitochondria. Both ubiquitin-dependent [47, 48] and ubiquitin-independent mitophagy [51] have been observed in the removal process. As the SPATA33-mediated mitophagy is ubiquitin-independent, whether SPATA33-mediated mitophagy is involved in paternal mitochondria removal in mammals remains an open question.

In summary, we identify a new kind of autophagy mediator SPATA33 in germline, which promotes mitophagy as well. The SPATA33-mediated mitophagy broadens our understanding of selective autophagy and mitochondrial homeostasis.

## Materials and methods

### Animals

Mouse strain ICR was purchased from the Wuhan Disease Prevention Center. The strain Kunming White was purchased from the Animal Center of the Wuhan Zhongnan Hospital. All animals were raised in the animal center with P3 grade in Wuhan University. The research is conducted in accordance with the guiding principles for biomedical research involving animals of Ethics and Animal Welfare Committee of College of Life Sciences of Wuhan University and the committee.

### Antibodies and reagents

Primary antibodies: Anti-SPATA33 was prepared by Wuhan Virus Research Institute of CAS, Wuhan, China. Anti-LC3B (3868, Cell Signaling Technology, Danvers, USA), Anti-ATG16L1 (8089, Cell Signaling Technology), Anti-GAPDH (CW0100, CWBIO, Beijing, China), Anti-FLAG (F3165, Sigma-Aldrich, St Louis, USA), Anti-MYC (11667149001, Roche Applied Science, Indianapolis, USA), Anti-GFP (11814460001, Roche Applied Science), Anti-VDAC2 (11663-1-AP, Proteintech Group, Rosemont, USA), Anti-SP1 (ab13370, Abcam Inc., Cambridge, USA), Rabbit Polyclonal ATG16L1 Antibody [Alexa Fluor^®^488] (NB110-60928AF488, NOVUS, Beijing, China), Rabbit Polyclonal LC3B Antibody [Alexa Fluor^®^488] (NB100-2220AF488, NOVUS, Beijing, China), COX-IV Monoclonal Antibody (YM0162, ImmunoWay, USA), Anti-SQSTM1 (A19700, Abclonal, Wuhan, China).

Secondary antibodies: Peroxidase-conjugated AffiniPure goat anti-mouse IgG, light chain specific (115-035-174, Jackson ImmunoResearch Laboratories, West Grove, USA), Peroxidase-conjugated AffiniPure fragment rabbit anti-mouse IgG, Fc fragment specific (315-036-046, Jackson ImmunoResearch Laboratories), AMCA-conjugated AffiniPure goat anti-mouse IgG (H + L) (31430, Pierce Biotechnology Company, Carlsbad, USA), Goat anti-rabbit IgG horseradish peroxidase (HRP)-linked whole antibody (31460, Pierce Biotechnology Company). Fluorescein antibodies: FITC-conjugated ImmunoPure goat anti-rabbit IgG (H + L) (ZF-0311, Zhongshan, Beijing, China), TRITC-conjugated ImmunoPure goat anti-rabbit IgG (H + L) (ZF-0316, Zhongshan), and Dylight 405 Donkey anti-rabbit IgG (H + L) (ANT081, Antgene, Wuhan, China).

Reagents: MitoTracker^®^ Red CMXRos (167095-09-2, YESEN, Shanghai, China), CCCP (T7081, TargetMol, Shanghai, China), and Bafilomycin A1 (B1793, Sigma-Aldrich, USA).

### Plasmid constructs and gRNA design

Full-length SPATA33 (NM_177279.4) was cloned into pCMV-Tag2B-3xFLAG, pcDNA3.0-MYC, and pSico-Cherry-FLAG using *Eco*RI and *Xho*I to generate p3xFLAG-SPATA33, pMYC-SPATA33, and pCherry-FLAG-SPATA33, respectively, while pSico-GFP-SPATA33 was made by *Xho*I and *Eco*RI digestions. Full-length SPATA33, N-60aa-SPATA33, and C-73aa-SPATA33 were cloned into pEGFP-N1 using *Xho*I and *Eco*RI to generate pGFP-SPATA33, pGFP-SPATA33-N, and pGFP-SPATA33-C. Full-length SPATA33, N-60aa-SPATA33, and C-73aa-SPATA33 were cloned into plove-CMV using *Xho*I and *Eco*RI to generate plove-SPATA33, plove-SPATA33-N, and plove-SPATA33-C. Full-length ATG16L1 (NM_001205391.1), N-306aa-ATG16L1, and C-317aa-ATG16L1 were digested with *Sal*I and *Hind*III, while the pSico-Cherry-FLAG and pEGFP-N1 were digested with *Xho*I and *Hind*III, and were cloned to generate pCherry-FLAG-ATG16L1, pGFP-ATG16L1-N, and pGFP-ATG16L1-C, respectively. 101st to 152nd amino acids in *Fis1* (NM_025562.3) were cloned into pmCherry-GFP-LC3B-m (P0202, MiaoLing Plasmid Sharing Platform, Wuhan, China) using *Hind*III and *Mfe*I to generate pmCherry-GFP-FIS1^101-152^.

LentiCRISPRv2-SPATA33-gRNA and LentiCRISPRv2-ATG16L1-gRNA were constructed as described previously [59, 60]. Briefly, SPATA33-gRNAs and ATG16L1-gRNAs were designed according to CRISPR Design Tool (http://crispr.mit.edu/) and synthesized with *Bsm*BI sticky end, then annealed and inserted into the lentiCRISPRv2 plasmid digested with *Bsm*BI (Fermentas, Vilnius, Lithuania). p3xFLAG-VDAC2 and pGFP-VDAC2 constructs were constructed as our previous study [61]. All the primers are described in Table S1.

### Cell culture and transfection

HEK293T, TM4, GC-1, and HeLa cells obtained from China Center for Type Culture Collection. The cells were cultured in high glucose DMEM (SH30022.01B, HyClone, Logan, USA) with 12% FBS (P30-330250, PAN-Biotech, Aidenbach, Germany) at 37 °C in a 5% CO_2_ in cell incubator. For transfection, cells were cultured in cell plates or glass cover slides. Lipofectamine 2000 (11668027, Invitrogen) was used in each well. For starvation treatments, the cells were cultured in EBSS (Cat# SH30029.02, HyClone, Logan, USA) for various times.

### Western blot analysis and coimmunoprecipitation assays

Western blot analysis was performed using routine protocols. Protein extracts from cells were separated in 12% SDS-polyacrylamide gels and then transferred onto 0.45-μm membranes (NK0414, Roche Diagnostics, Indianapolis, IN, USA). Primary antibodies were incubated with the membranes overnight at 4 °C. The membranes were washed in TBST (20 mM Tris-HCl pH7.5, 150 mM NaCl, 0.1% Tween 20) three times, incubated with the indicated HRP-conjugated secondary antibody for 1 h at room temperature, and then washed in TBST five times. A Super Signal Chemiluminescent Substrate system (K-12045-D50, Advansta, Menlo Park, USA) was used to detect the signals.

Coimmunoprecipitation was used to analyze protein interactions in vitro. HEK293T cells were co-transfected with related plasmid DNAs. After 48 h, the cells were lysed in IP buffer (50 mM Tris-HCl at pH 8.0, 0.15 M NaCl, 1 mM EGTA, and 0.5% NP-40) containing protease inhibitor cocktail (04693159001, Roche Applied Science, Indianapolis, USA). The other steps are described in the previous study [62].

### Immunofluorescence analysis

Testis tissues were embedded in OCT medium (4583, Tissue-Tek, Miles, USA) and cut into a series of 8 μm sections using a cryostat (Leica, Bensheim, Germany). GC-1, TM4 and HeLa cells were cultured on glass cover slides. Both sections and cover slides were fixed with 4% PFA for 20 min at room temperature, then permeabilized with 1% Triton X-100 (0.1% Triton for cell lines) (9002-93-1, Sigma-Aldrich) in PBS for 30 min. The other steps were described in the previous study [56]. Images were taken by confocal fluorescence microscopy (SP8, Leica).

### MitoQC-based assays

GC-1 cells (wild type, *Spata33*-overexpressed, and *Spata33* KO cell lines) were cultured on cover slides and transfected with the pmCherry-GFP-FIS1^101-152^. After 24 h in normal culture, the cells were treated with normal medium (control), CCCP (10 μM, 1 h), EBSS medium (1 h), and EBSS with CCCP (10 μM, 1 h) addition, respectively. The other steps were described in the immunofluorescence analysis. Images were taken by confocal fluorescence microscopy (SP8, Leica).

### Flow cytometry

Testicular tissue was removed under aseptic conditions from adult male mice and placed in a petri dish containing precooled PBS. Testicular white membrane was removed with pointed tweezers under sterile conditions, and the samples were shred in another clean petri dish within 0.25% trypsin and 0.2% collagenase I, digested with shaking in 37 °C incubator for 10–20 min. After that, the digestion was stopped by adding 4 ml DMEM/F12 medium and 20% FBS medium. The sample were blown and beaten repeatedly until the cells dispersed, and passed through a 70 μm filter into a new centrifuge tube, then centrifuged at 1800 rpm for 10 min at room temperature. After supernatant discarded, precipitation was resuspended in 4 ml medium with 20% FBS and penicillin-streptomycin. Flow cytometry (SH800S, SONY, Japan) was used for sorting based on the physical properties of the cells (the size and granule density of the cells).

### Semi-quantitative RT-PCR

TRIzol (15596-026, Invitrogen) was used to isolate total RNA, which was transcribed using a poly (T)18 primer and MMLV (M1701, Promega). Specific primers were designed to amplify the *Spata33* gene by PCR instrument (S1000^TM^ Thermal Cycler, BIO-RAD, California, USA). *Actin* was amplified as a control. The primer sequences are described in Table S1.

### Lentivirus generation, infection, and gene knockout

To generate lentivirus as described before, the HEK293T cells seeded on 100-mm plate were transfected with lentiCRISPRv2-SPATA33-gRNA and lentiviral packaging vectors pRSV-Rev (12253, Addgene, Watertown, USA), pMD2.G (12259, Addgene), and pCMV-VSV-G (8454, Addgene) using Lipofectamine 2000 according to the manufacturer’s instructions. After incubation for 48 h, the supernatants were filtered through 0.45 μm filters and used directly to infect TM4 or GC-1 cells. Puromycin (A1113802, Fisher Scientific, Waltham, USA) was used to screen the cells. The primer sequences are described in Table S1.

### Transmission electron microscope

After treated with starvation and CCCP (10 μM) for 1 h, GC-1 cells (WT and *Spata33*-overexpressed) were fixed with 3% PFA, 1.5% glutaraldehyde, and 2.5% sucrose, then washed with PB buffer (19 ml: 0.2 M NaH_2_PO_4_; 81 ml: 0.2 M Na_2_HPO_4_) for 3 times, each time for 5 min. The cells were collected by gradient centrifugation at 4 °C, and immobilized with osmic acid in ice bath for 1 h. The cells were stained overnight with uranium dioxy acetate, and gradient dehydration was followed. The resin was embedded and hardened, then the samples were sectioned at a thickness of 70 nm using an ultramicrotome (EM UC7, Leica) and observed by transmission electron microscope (Tecnai G^2^ 20, FEI, Oregon, USA).

## Supplementary information

Supplementary figure legends

Supplementary Figure 1

Supplementary Figure 2

Supplementary Figure 3

Supplementary Figure 4

Supplementary Figure 5

Supplementary Figure 6

Supplementary methods and tables
