# Supplementary material for: SPATA33 is an autophagy mediator for cargo selectivity in germline mitophagy
Source: Cell Death Differ. 2020 Oct 21;28(3):1076–90. doi: 10.1038/s41418-020-00638-2 (PMC7937689; doi:10.1038/s41418-020-00638-2)
Supplement: Supplementary file 1 — Supplementary figure legends [file 41418_2020_638_MOESM1_ESM.docx]

**Supplementary figure legends**

**Fig. S1.** SP1 activates *Spata33* gene promoter in both vitro and in vivo.

(**a**) Schematic diagram of the *Spata33* promoter. Three binding sites of SP1 (a, b, and c) were detected in the promoter. The sequences of the binding sites are showed on the left panel.

(**b**) Luciferase assay showed the activity of a series of truncated constructs in HEK293T cells. Left panel indicates each deleted mutant linked with the luciferase gene in the pGL3-basic vector. Right panel shows the relative activity of these deleted constructs, as determined by luciferase assays. The results were representative of 3 independent experiments and represented as means ± SD. The data were analyzed using Student’s *t*-test. **, *p*< 0.01.

(**c**) *Spata33* and *Sp1* expression in postnatal testis of mice by semi-quantitative RT-PCR. *Actin* was used as an internal control.

(**d**) *Spata33* promoter and fused structure with luciferase gene for activity measurement.

(**e**) Point mutation analysis of the promoter using luciferase assays in HEK293T cells. The LS4 construct of 135 bp was used as a basic construct for the point mutation analysis. The intact binding sites of SP1 are donated by circles. The filled circles show corresponding mutations. The pGL3-basic vector was used as a negative control. Statistical analysis was performed as in panel B. *, *p*< 0.05; **, *p*< 0.01.

(**f**) Overexpression of SP1 activated the *Spata33* promoter in HEK293T cells. pGL3-LS4 or *Sp1* site mutants (pGL3-LS4-SP1mut1, pGL3-LS4-SP1mut2, and pGL3-LS4-SP1mut3) were co-transfected with *Sp1* expression plasmid (pCMV-SP1) as indicated. SP1 overexpression activated the activities of all the constructs except pGL3-LS4-SP1mut3. Statistical analysis was performed as in panel B. *, *p*< 0.05; **, *p*< 0.01.

(**g**) ChIP assay. Chromatin immunoprecipitation analysis was performed from sonicated chromatin of testis samples of adult mice. The samples were immunoprecipitated with no antibody (beads only), preimmune IgG (control) or anti-SP1. The fragment of 136 bp corresponding to the -77 bp to +62 bp region of the *Spata33* promoter was amplified using the immunoprecipitated DNA as a template. Intron 2 of *Spata33* was used as a negative control.

(**h**) Schematic diagram of primer positions in the ChIP assay.

**Figure S2**. Targeted disruption of *Spata33* using CRISPR/Cas9 system in TM4 cell line.

(**a**) Schematic representation of gRNAs targeting the *Spata33* locus. The translation start codon ATG is indicated by an arrow, and exons are numbered from 1 to 3. Two gRNAs (blue) are designed to exon 2. PAMs, protospacer adjacent motif, are highlighted in red.

(**b**) PCR analysis of genomic DNA isolated from monoclonal cells. Primer sequences and PCR conditions are listed in Table S1.

(**c**) Nucleotide sequence alignments of *Spata33* mutant alleles of clone #32-5, #32-10 and #29-5 with WT sequence. The deleted sequences are indicated in dash lines.

(**d**) Schematic diagram of protein coding regions of wide type SPATA33 and predicted truncated mutants. The coding region of three exons are indicated in boxes (solid lines). Frameshift sequences are shown in boxes with dash lines. The numbers refer to the amino acid positions.

**Figure S3**. Targeted disruption of *Spata33* using CRISPR/Cas9 system in GC-1 cell line.

(**a**) Schematic representation of gRNAs targeting the *Spata33* locus. The translation start codon ATG is indicated by an arrow and exons are numbered from 1 to 3. Two gRNAs (blue) are designed to target exon 1. PAMs, protospacer adjacent motif, are highlighted in red.

(**b**) PCR analysis of genomic DNA isolated from monoclonal cells. Primer sequences and PCR conditions are listed in Table S1.

(**c**) Nucleotide sequence alignments of *Spata33* mutant alleles of clone #24, #29 and #33 with WT sequence. The deleted sequences are indicated in dash lines.

(**d**) Schematic diagram of protein coding regions of wide type SPATA33 and predicted truncated mutants. The coding region of three exons are indicated in boxes (solid lines). Frameshift sequences are shown in boxes with dash lines. The numbers refer to the amino acid positions.

**Figure S4.** Off-target analysis in TM4 cell line (#32-10).

(**a**) The predicted off-target sites was aligned with TM4 cell line genome.

(**b**) PCR amplification of predicted off-target sequences. The PCR products were then cloned into pGEM-T Easy vector and sequenced. The off-target sites were underlined in black.

**Figure S5.** Off-target analysis in GC-1 cell line (#33).

(**a**) The predicted off-target sites was aligned with GC-1 cell line genome.

(**b**) PCR amplification of predicted off-target sequences. The PCR products were then cloned into pGEM-T Easy vector and sequenced. The off-target sites were underlined in black.

**Figure S6.**

(**a**) Colocalization analysis of SPATA33 with VDAC2 and ATG16L1 in HeLa cells. The cells were transiently co-transfected with pCherry-SPATA33 and pGFP-VDAC2. After grown in normal medium for 24 h, the cells were cultured in normal (control), CCCP (100 μM, 2 h), EBSS medium (2 h) or EBSS with CCCP (100 μM, 2 h) addition, respectively. Immunofluorescence analysis were performed with anti-ATG16L1 and Dylight405 Donkey anti Rabbit IgG (H+L) antibodies. Single channel (red, green or blue) and merged images were taken by confocal microscopy. Colocalizing structures are indicated in white (merge). Scale bar: 25 μm.

(**b**) Statistical analysis of colocalized puncta between SPATA33, ATG16L1 and VDAC2. Data are presented as means ± S.D. **, *p*< 0.01 (n = 3 independent experiments, >15 cells per experiment).
