# Supplementary figures and images for: SPATA33 is an autophagy mediator for cargo selectivity in germline mitophagy
Source: Cell Death Differ. 2020 Oct 21;28(3):1076–90. doi: 10.1038/s41418-020-00638-2 (PMC7937689; doi:10.1038/s41418-020-00638-2)

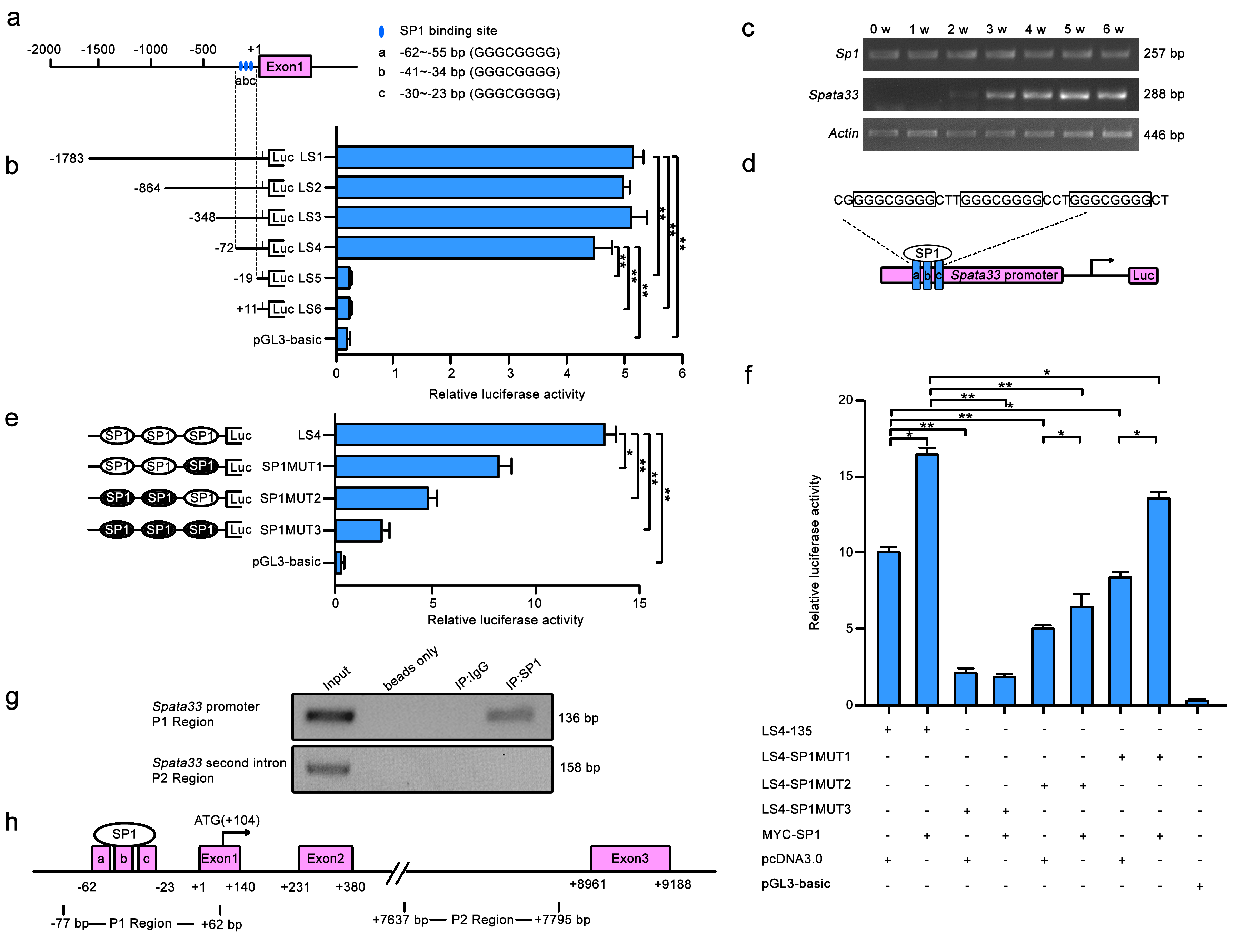

Supplement: Supplementary file 2 — Supplementary Figure 1 [file 41418_2020_638_MOESM2_ESM.png]

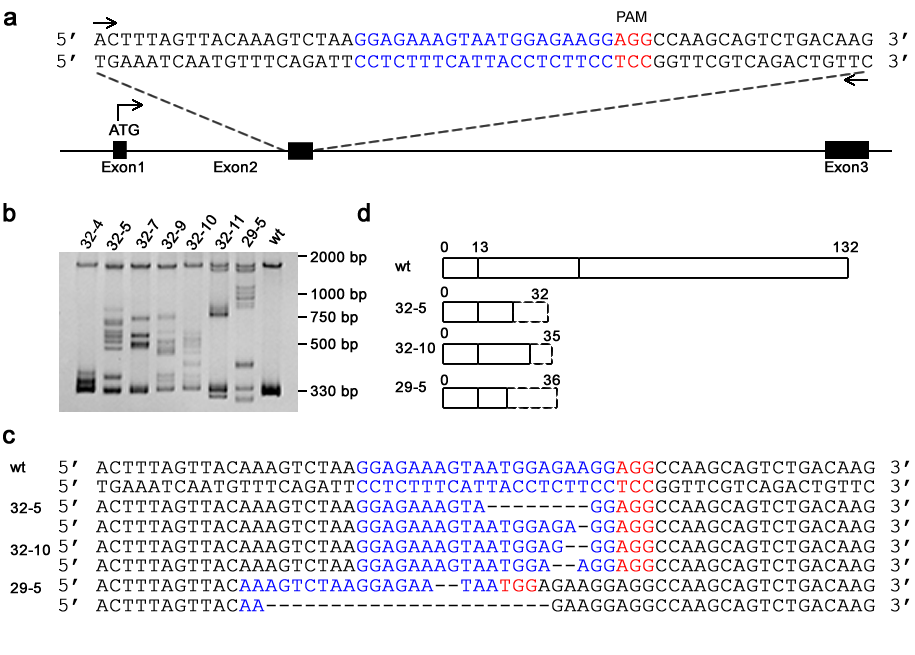

Supplement: Supplementary file 3 — Supplementary Figure 2 [file 41418_2020_638_MOESM3_ESM.png]

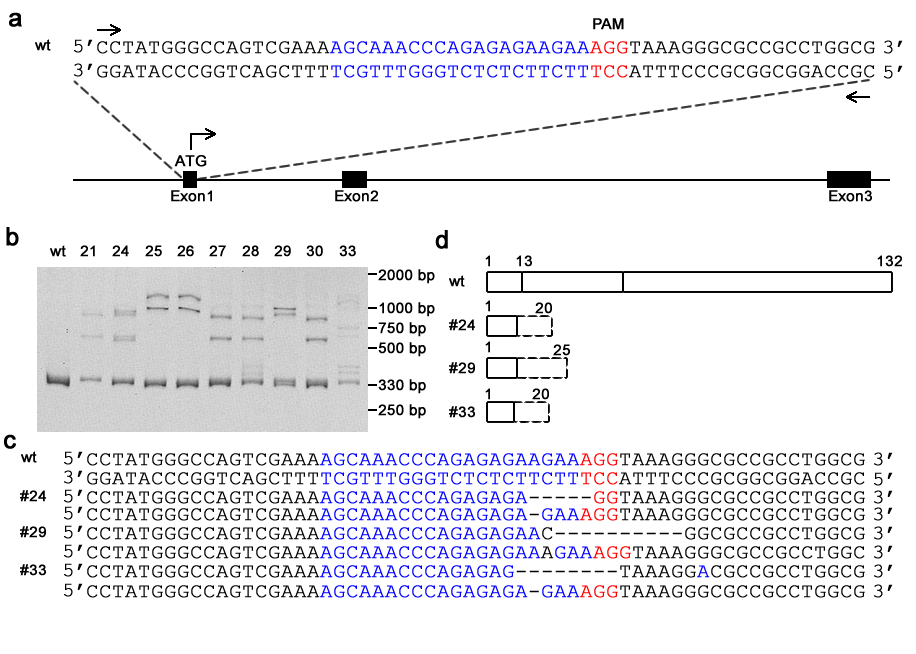

Supplement: Supplementary file 4 — Supplementary Figure 3 [file 41418_2020_638_MOESM4_ESM.png]

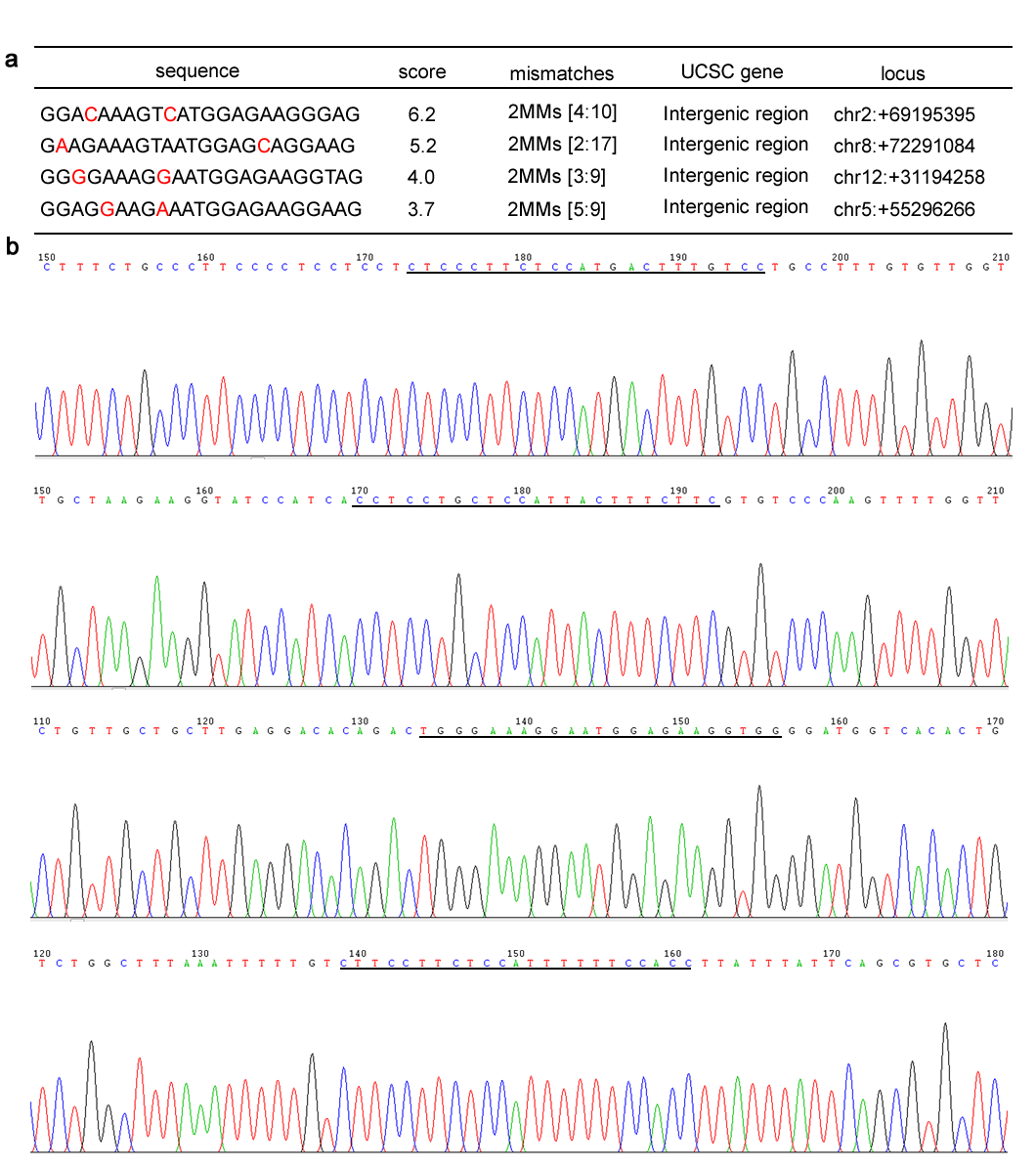

Supplement: Supplementary file 5 — Supplementary Figure 4 [file 41418_2020_638_MOESM5_ESM.png]

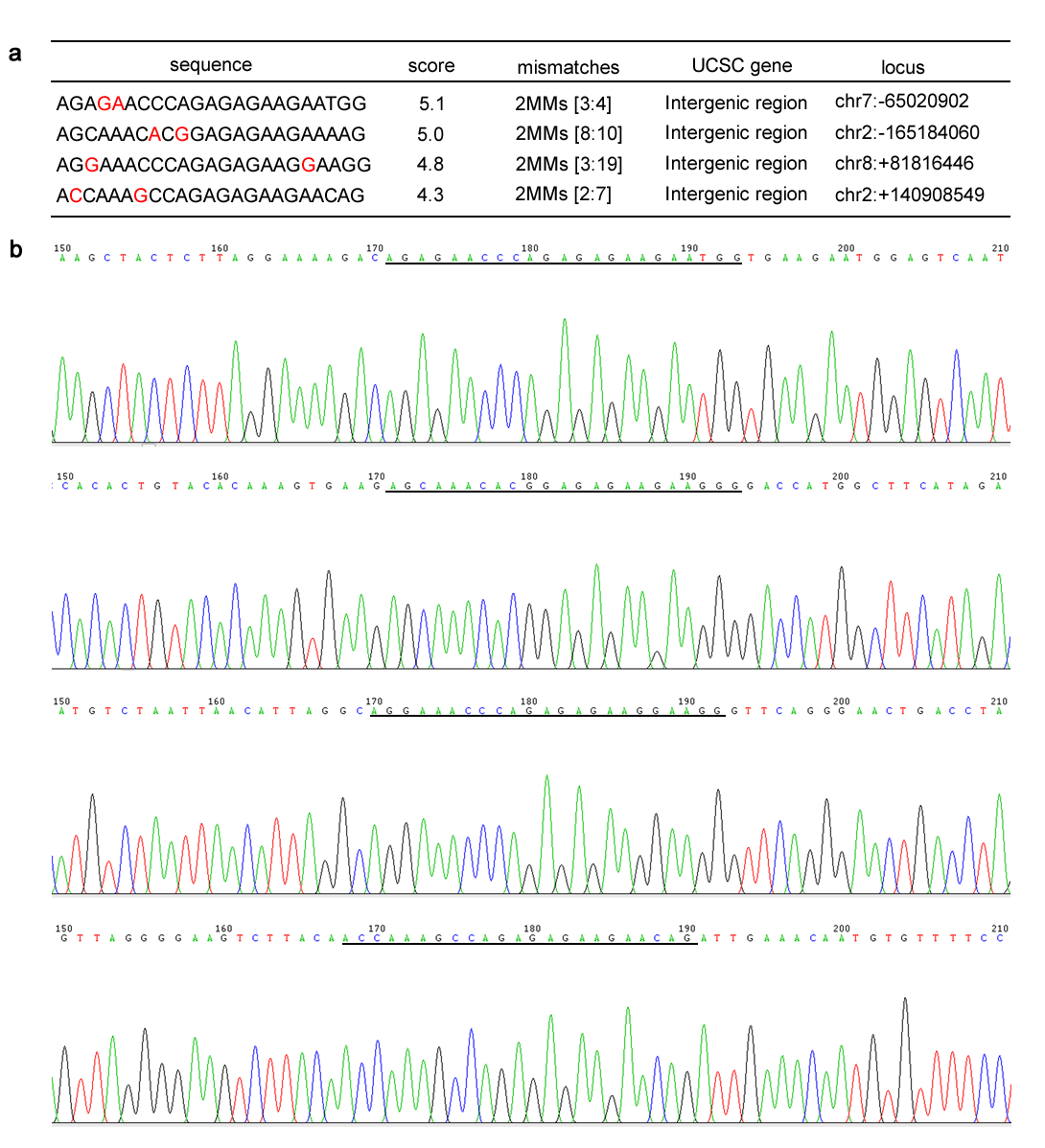

Supplement: Supplementary file 6 — Supplementary Figure 5 [file 41418_2020_638_MOESM6_ESM.png]

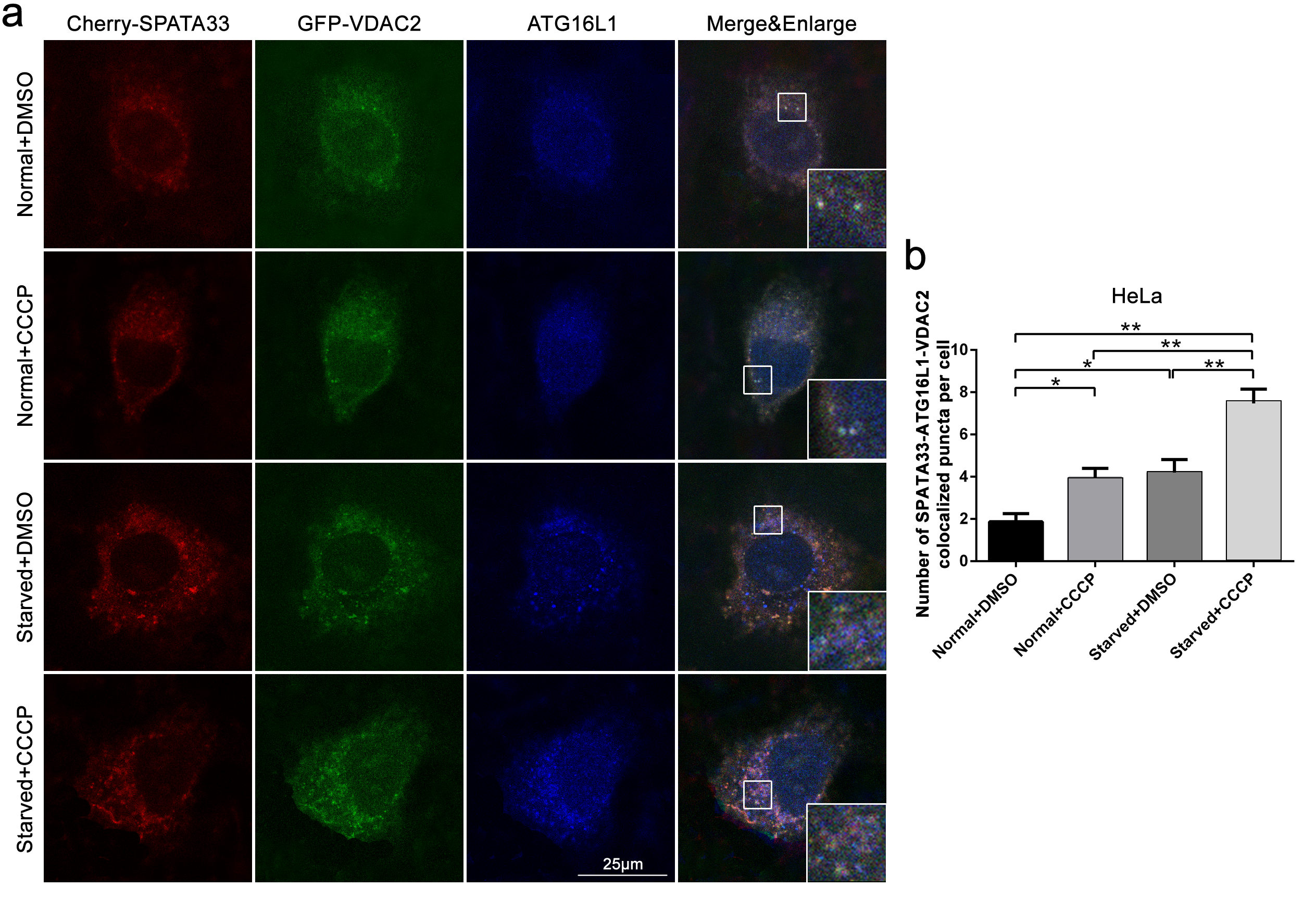

Supplement: Supplementary file 7 — Supplementary Figure 6 [file 41418_2020_638_MOESM7_ESM.png]
