# Supplementary material for: SPATA33 is an autophagy mediator for cargo selectivity in germline mitophagy
Source: Cell Death Differ. 2020 Oct 21;28(3):1076–90. doi: 10.1038/s41418-020-00638-2 (PMC7937689; doi:10.1038/s41418-020-00638-2)
Supplement: Supplementary file 8 — Supplementary methods and tables [file 41418_2020_638_MOESM8_ESM.docx]

**­ Supplementary methods and tables**

**Supplementary methods**

**Plasmid constructs**

Eleven deletion fragments of the mouse *Spata33* promoter were amplified from mouse genomic DNA, which were double-digested with *Mlu*I and *Hind*III and cloned into the pGL3-basic vector (E1751, Promega, Madison, USA). Transcription factor SP1 expression plasmid (MYC-SP1) was from our previous research. Site-directed mutagenesis for the SP1(SP1MUT1 and SP1MUT2) binding sites were performed using the primers described in Table S1. SP1MUT1 was used as a template for constructing SP1MUT3.

**Cell culture, transfection, and dual-luciferase reporter assays**

HEK293T cells were obtained from China Center for Type Culture Collection. The cells were cultured in high glucose DMEM (SH30022.01B, HyClone, Logan, USA) with 12% FBS (P30-330250, PAN-Biotech, Aidenbach, Germany) in 48-well plates. For transfection, Lipofectamine 2000 was used in each well. For luciferase assays, each WT or deletion construct was transfected at 500 ng, together with 5 ng/well of pRL-TK (E2241, Promega, Madison, USA). After transfection, Luciferase activities were measured using a dual-luciferase reporter assay system (Promega, Madison, WI, USA) and a Modulus Single Tube Multimode Reader (Turner Biosystems, Sunnyvale, CA, USA). Experiments were independently repeated at least 3 times.

**Chromatin immunoprecipitation**

Mouse testis were cut into small pieces, and 1% formaldehyde-PBS was used for cross-linking for 15 min with constant shaking. Then, glycine was added to a final concentration of 0.125 M to terminate the crosslinking. The other steps were described in the previous study. The supernatant fraction was immunoprecipitated with anti-SP1, beads or pre-immune IgG together with protein G PLUS-agarose (Sc-2002, Santa Cruz, USA). DNA from the immunoprecipitated complex was PCR-amplified using primers flanking -77 to +62 bp of the *Spata33* genomic sequence. As a control, a 159-bp of *Spata33* region approximately 7 kb downstream of intron 2 of the *Spata33* was amplified. The PCR fragments were cloned into the T-easy vector (A137A, Promega) and sequenced. The primer sequences are described in Table S1.

**Off-target assays**

CRISPR Design Tool was used to predict the potential off-target sites (http://crispr.mit.edu/). All possible off-target sites were screened by ungapped alignments, allowing for up to four mismatches in the target gRNA sequence. In the output, four potential off-target sequences with high scores for every gRNA were selected and PCR amplified using genomic DNA from TM4 or GC-1 as templates. The PCR products were then cloned into pGEM-T Easy vector (A1360, Promega) and sequenced. At least 5 recombinant clones were sequenced. All the primers are described in Table S1.

**­**

**Supplementary tables**

**Table S1. The primers used in the study**

| **Construct name Primer sequences (5′-3′)** | |
| --- | --- |
| pGL3-LS1 | F: ATAACGCGTACACTTTGACCTCTTGACCCTT  R: ATAAAGCTTTAACCCGACCGACTCACAAA |
| pGL3-LS2 | F: ATAACGCGTACTCATACTGCTACGGGTGGTT  R: ATAAAGCTTTAACCCGACCGACTCACAAA |
| pGL3-LS3 | F: ATAACGCGTGGTCCCGGTCCGAATACA  R: ATAAAGCTTTAACCCGACCGACTCACAAA |
| pGL3-LS4 | F: ATAACGCGTTGTCGCGAGAAGATAGCTCC  R: ATAAAGCTTTAACCCGACCGACTCACAAA |
| pGL3-LS5 | F: ATAACGCGTCAGGCACGGCTCGCGCGGCA  R: ATAAAGCTTTAACCCGACCGACTCACAAA |
| pGL3-LS6 | F: ATAACGCGTATGGTAATGAGCTCCCGGAG  R: ATAAAGCTTTAACCCGACCGACTCACAAA |
| pGL3-SP1-mut1 | F: CGGGGCGGGGCTTGGGCGGGGCCTGCCGCCGGCTCAGGCACGGCT  R: AGCCGTGCCTGAGCCGGCGGCAGGCCCCGCCCAAGCCCCGCCCCG |
| pGL3-SP1-mut2 | F: CGGCCGCCGGCTTGCCGCCGGCCTGGGCGGGGCTCAGGCACGGCT  R: AGCCGTGCCTGAGCCCCGCCCAGGCCGGCGGCAAGCCGGCGGCCG |
| pGL3-SP1-mut3 | F: CGGCCGCCGGCTTGCCGCCGGCCTGCCGCCGGCTCAGGCACGGC  R: GCCGTGCCTGAGCCGGCGGCAGGCCGGCGGCAAGCCGGCGGCCG |
| psi-3x-FLAG-SPATA33 | F: AATGAATTCATGGGCCAGTCGAAAAGCAA  R: ATTCTCGAGTTCTGTGTTGTGTACATCAT |
| MYC-SPATA33 | F: AATGAATTCATGGGCCAGTCGAAAAGCAA  R: ATTCTCGAGTTCTGTGTTGTGTACATCAT |
| Cherry-FLAG-SPATA33 | F: AATGAATTCATGGGCCAGTCGAAAAGCAA  R: ATTCTCGAGTTCTGTGTTGTGTACATCAT |
| SPATA33-EGFP | F: AATCTCGAGATGGGCCAGTCGAAAAGCAA  R: AATGAATTCGTTCTGTGTTGTGTACATCAT |
| psi-GFP-SPATA33 | F: ATACTCGAGATGGGCCAGTCGAAAAGCAA  R: AATGGATCCCGTTCTGTGTTGTGTACATCATAGGC |
| Cherry-FLAG-ATG16L1 | F: AATGTCGACATGTCGTCGGGCCTGCGCGC  R: AATAAGCTTAGGCTGTGCCCACAGCACAG |
| ATG16L1-EGFP | F: AATGTCGACATGTCGTCGGGCCTGCGCGC  R: AATAAGCTTAGGCTGTGCCCACAGCACAG |
| ATG16L1-N-EGFP | F: AATGTCGACATGTCGTCGGGCCTGCGCGC  R: AATAAGCTTGGAAGAGACAGAGCGTCTCC |
| ATG16L1-C-EGFP | F: AATGTCGACATCCCAGTCCCCCAGGATAT  R: AATAAGCTTAGGCTGTGCCCACAGCACAG |
| SPATA33-N-EGFP | F: AATCTCGAGATGGGCCAGTCGAAAAGCAA  R: AATGAATTCGCGAAGACGACGGCCTTGAGT |
| SPATA33-C-EGFP | F: AATCTCGAGTCTGAAGACAAACCTGAGAC  R: AATGAATTCGTTCTGTGTTGTGTACATCAT |
| Plove-SPATA33 | F: AATCTCGAGATGGGCCAGTCGAAAAGCAA  R: AATGAATTCTTCTGTGTTGTGTACATCAT |
| Plove-N-SPATA33 | F: AATCTCGAGATGGGCCAGTCGAAAAGCAA  R: AATGAATTCCGAAGACGACGGCCTTGAGT |
| Plove-C-SPATA33 | F: AATGAATTCCGAAGACGACGGCCTTGAGT  R: AATGAATTCTTCTGTGTTGTGTACATCAT |
| Fis1 | F: AAT AAGCTT GAGCCCCAGAACAACCAGGC  R: AAT CAATTG GGATTTGGACTTGGAGACAG |

**The Primers used in Semi-quantitative PCR analysis**

| **Name** **Primer sequences (5′-3′)** | |
| --- | --- |
| Spata33 | F: AGGAGGAGAAGAGCACTACCA  R: CATGCTCCCGAATGGTTCTCT |
| β-Actin | F: GAGACCTTCAACACCCCAGC  R: CCACAGGATTCCATACCCAA |
| Sp1 | F: GCTACCCCTACCTCAAAGGAA  R: ATTTGACCAGAACCATCCTGC |

**The primers used in ChIP**

| **Name** **Primer sequences (5′-3′)** | |
| --- | --- |
| CHIP | F: CTGTCGCGAGAAGATAGCTCC  R: TAACCCGACCGACTCACAAAA |
| CHIP-control | F: CTACACAGAGAAACCCTGTCT  R: AAGTGCCTTTGCCTCTTGAGA |

**The Primers used in gene knockout**

| **Name** **Primer sequences (5′-3′)** | |
| --- | --- |
| SPATA33-11AA | F: CACCGAGCAAACCCAGAGAGAAGAA  R: AAACTTCTTCTCTCTGGGTTTGCTC |
| SPATA33-29AA | F: CACCGAAAGTCTAAGGAGAAAGTAA  R: AAACTTACTTTCTCCTTAGACTTTC |
| SPATA33-32AA | F: CACCGGGAGAAAGTAATGGAGAAGG  R: AAACCCTTCTCCATTACTTTCTCCC |
| Screening | F: ATGGTAATGAGCTCCCGGAG  R: GAACAACAGGCTTTCGGCAG |
| ­­ATG16L1-1 | F: CACCGCTGGAAGCGTCACATCGCGG  R: AAACCCGCGATGTGACGCTTCCAGC |
| ATG16L1-2 | F: CACCGGCGCCGCAGACTTTCCCCGC  R: AAACGCGGGGAAAGTCTGCGGCGCC |
| ATG16L1-3 | F: CACCGGGCGCCGGGACCGACTGCAG  R: AAACCTGCAGTCGGTCCCGGCGCCC |

**The Primers used in off-target assay**

| **Name** **Primer sequences (5′-3′)** | |
| --- | --- |
| GC1-2 | F: TTTTAATGGTCATGTCTCCAC  R: ATGAAGAGTTGCTAAGTCACT |
| GC1-3 | F: ATTGGCAAAAGCTGTTCCAAC  R: CCACAGTGTCTTATGTGTGAT |
| GC1-4 | F: ACCATCATGGCCCTTCACATG  R: GACAGTGGTGCTCTTGAATGG |
| GC1-5 | F: TGCATATCATGTTGAAACCCA  R: AGACCAAATCAATTCTACCCC |
| TM4-1 | F: TTGGAATGTCGGAGTTATGTG  R: TTTCTGCTCCATCTTGCACTG |
| TM4-2 | F: TGCAGAAAGCTGAAGTTATGC  R: CCTAGGACAGCAACTCTCATG |
| TM4-4 | F: AGGGAGATGATGTCCCAACTG  R: GATGTTTCTTGGCAGGTTCTT |
| TM4-5 | F: CTACTCTCTACAGTGCCCTCC  R: AACTGAAGAAAGTGTGCCTCG |
